# Primary Carcinoid Tumour of the Kidney: A Review of the Literature

**DOI:** 10.1155/2013/579396

**Published:** 2013-08-13

**Authors:** Ayodeji O. Omiyale, Anthony Kodzo-Grey Venyo

**Affiliations:** ^1^Department of ENT Surgery, North Manchester General Hospital, Delaunays Road, Manchester, UK; ^2^Department of Urology, North Manchester General Hospital, Delaunays Road, Manchester, UK

## Abstract

*Context*. Primary renal carcinoid tumours are rare. Their pathogenesis is unknown and the clinical presentation is similar to other renal tumours thus posing diagnostic dilemmas for clinicians. *Objectives*. To review the literature for case reports of primary renal carcinoids. *Methods*. Literature was extensively searched for case reports for primary renal carcinoids. Reports of metastatic carcinoids to the kidneys were excluded. *Results*. Approximately less than 90 cases of primary carcinoid tumours of the kidney have been reported in the literature. A total of 29 cases of primary renal carcinoids were reviewed. The mean age of presentation was 48 years (range 29–75) with both right kidney (48.3%) and left kidney (44.8%) being equally affected. 28.6% of the cases reviewed were diagnosed as an incidental finding. The mean followup time was 20 months with 73.1% of patients without evidence of disease after surgical treatment (radical or partial nephrectomy). Primary carcinoid tumours of the kidney are often well differentiated tumours. They are often misdiagnosed because of their rarity and similar presentation with other renal tumours. *Conclusions*. Primary carcinoid tumours of the kidney are rare tumours with an indolent course with frequent metastasis. Metastatic work up and followup is required in their management.

## 1. Introduction

The histology of carcinoid tumours was first described by Lubarsch in 1888 [1], while the first case of carcinoid in the gastrointestinal tract was reported by Oberndorfer in 1907 [2].

Carcinoid tumours are neoplasms with neuroendocrine differentiation. They are thought to arise from APUD cells with characteristic secretory granules. Although they have certain clinicopathological features which are specific to the organ in which they arise, there are several features common to neuroendocrine tumours (NETs) regardless of the site of origin in the body [3].

There are various systems of nomenclature in the classification of NETs which has led to confusing terminologies. Some of which differ in the use of terminologies and criteria for staging and grading [4]. Attempts have been made to ensure a single, uniform, and reproducible system of nomenclature. Klimstra et al. in a review of the nomenclature, staging and grading systems in the pathologic classification of NETs however suggested that in all the systems a sharp distinction is made between well differentiated and poorly differentiated with the latter clearly described as high grade neuroendocrine cancers [3].

WHO in 2010 classified neuroendocrine neoplasms into NET-well differentiated grade 1, NET-well differentiated grade 2, NEC-poorly differentiated grade 3 (small and large cell type), mixed adenoneuroendocrine carcinoma (MANEC), hyperplastic and preneoplastic lesions. The first three however refer to carcinoid neoplasms [5].

NETs occur in various sites of the body; however, the gastrointestinal tract accounts for 73.7% while the respiratory tract accounts for 25.1% of carcinoid tumours [6]. They are known to occur rarely in the genitourinary system. In males, of all genitourinary carcinoids reported, the testis and prostate account for 55% and 15%, respectively, while the kidney and bladder account for 19% and 9%, respectively [7].

This paper seeks to review the literature for primary carcinoid tumours of the kidney because they are very rare, often mistaken for other renal tumours, the histopathogenesis are uncertain, and much is not known about them.

## 2. Methods

The literature was searched extensively for primary carcinoid tumour of the kidneys using Medline and Ovid SP databases. Romero et al. [7] in their review of primary renal carcinoid tumours till 2006 documented 56 cases. The inclusion criteria for our review were relevant publications of primary carcinoid tumour of the kidney after the published reports by Romero et al. till date.

Carcinoid tumours of the kidney secondary to metastasis from other sites were excluded. All studies were assessed for epidemiological, histological, clinical, diagnostic, therapeutic, and prognostic data. The search terms used were primary renal carcinoid tumours, neuroendocrine tumours of the kidney, primary tumours of the kidney, and so forth. Further publications were identified by manually searching the references of relevant research papers.

## 3. Results

The clinical and pathologic data of the 29 cases reviewed and their clinical outcome are shown in Table 1.

## 4. Discussion

Primary NETs of the kidney are very rare. Fourty six years after the first case was reported in 1966 by Resnick et al. [33] to the best knowledge of the authors approximately less than 90 cases have been reported in the literature.

### 4.1. Epidemiology

Individuals between the ages of 13 and 79 years have been known to present with carcinoid tumour of the kidney [34]. The fifth and sixth decades of life are the peak age of incidence [7]. The mean age of presentation in our review was 48 years (range 29–75).

Romero et al. in an analysis of 56 cases of primary carcinoid tumours of the kidneys reported that the right kidney was more involved than the left (60.9% versus 39.1%) [7]. Our review however demonstrates equal affectation of the both right (48.3%) and left kidney (44.8%). This agrees with the findings of Krishnan et al. who reported that both right and left kidneys are equally affected [34].

The female sex (62%) was more affected than the male sex in our review. This is in contrast to the findings of Romero et al. which suggested that there is no sex predilection; however carcinoid tumours associated with horseshoe kidneys tend to have a male sex predilection (1.5 : 1) which could partly be explained to be due to the higher incidence of horseshoe kidneys in men [7, 35]. Familial predisposition has not been reported [36].

### 4.2. Pathogenesis

Intrinsic NE cells in normal kidneys have not been reported, and the pathogenesis of primary carcinoid tumour of the kidney is uncertain and remains largely unknown [34]. NE cells have been observed in the urothelium of the upper urinary tract [37–39].

Several theories regarding the origins of carcinoid tumours have been postulated. The first theory suggests origin from an intrinsic neuroendocrine cell either representing a minute endocrine-paracrine cell component or a misplaced or entrapped progenitor cell (neural crest) of the dispersed neuroendocrine system during embryogenesis [39]. Guy et al. in the immunohistochemical study of foetal, infantile, and adult kidneys concluded that no neuroendocrine cell was identified in the renal parenchyma thus invalidating the theory of intrinsic NE cells in the pathogenesis of renal carcinoid tumour [39].

The theory of derivation from interspersed NE cells due to intestinal metaplasia of the pyelocalyceal urothelium induced by chronic infection or inflammation has been considered [34]. Romero et al. noted that intestinal metaplasia have not been frequently reported in these tumours and there are no evidence to suggest chronic pyelonephritis or infection [7]. Metastasis from unknown primary tumours to the kidneys is another plausible hypothesis [7].

The theory of stem cell neuroendocrine modulation through neoplastic activation of gene sequences common to neuroendocrine programmed cells or primitive multipotential cell lines that differentiate in a NE direction has been postulated [36, 37, 39, 40]. El-Naggar et al. noted loss of heterozygosity at 1 locus on chromosome 3p21. They concluded that 3p aberrations may constitute a predifferentiation tumour inducing event in the development of renal neoplasms including renal carcinoids [37].

### 4.3. Associated Pathologies

Primary renal carcinoids are known to be associated with some other renal pathologies. In a previous review of 56 cases, renal carcinoids were associated with horseshoe kidneys (17.8%), teratomas (14.3%), and polycystic kidney disease (1.8%) [7]. Our review demonstrates associations with horseshoe kidneys (25%), one case of teratoma and one case of mature teratoma coexisting with primary renal carcinoid tumour in a horseshoe kidney.

Horseshoe kidneys are associated with increased risk of Wilms tumour, neoplasms of the renal pelvis and carcinoid tumours of the kidney [34]. The relative risk of carcinoid tumours in individuals with horseshoe kidneys compared to individuals with normal kidneys ranges from 62 to 120 [34, 41, 42]. There are 8 cases of primary renal carcinoid tumour associated with horseshoe kidneys in our review [10, 13, 18, 26, 27, 29].

The reported frequent occurrence of renal carcinoids in the isthmus of the horseshoe kidneys lends credence to the notion that teratogenic events related to the abnormal migration of posterior nephrogenic cells in utero may play a role in the pathogenesis [34]. A case of synchronous adenocarcinoma and primary renal carcinoid tumour arising within a teratoma in a horseshoe kidney has been reported [10].

Teratomas occurring as primary renal tumours are quite rare. Teratomas have been reported to arise in association with primary renal carcinoid [12, 43–46]. It is however believed that the carcinoid tumours in these teratomas are derived from neuroendocrine cells which arise from gastrointestinal or respiratory teratoid epithelial cellular components [44]. Cases of primary renal carcinoids arising within mature teratoma in horseshoe kidneys have also been reported [47, 48].

Other associated pathologies include polycystic kidney disease. Primary renal carcinoid tumour was first reported by Shibata et al. [49] as an incidental finding in a lady with autosomal dominant adult polycystic kidney disease who underwent bilateral nephrectomy because of infections of her polycystic kidney. Mutations in PKD1 and PKD2 genes are responsible for the development of polycystic kidney disease. The possibility of these mutations is believed to be low in renal carcinoid tumours and the risk of development of primary carcinoid tumour in people with polycystic kidney disease is believed to be very low [49].

### 4.4. Clinical Presentation

The diagnosis of primary carcinoid tumour of the kidney has been reported to be incidental in 25–30% of cases [50]. This is similar to the findings of this review with primary carcinoid tumour of the kidney diagnosed as an incidental finding in 28.6% of cases [13, 18, 20, 23, 32]. The clinical presentation is similar to other renal neoplasms. The most commonly reported presenting complaint was abdominal or flank pain [9, 10, 12, 15–17, 19, 21, 22, 26–30]. Other symptoms include haematuria [8, 11, 18, 24] constipation, urinary frequency, fever, epigastric discomfort, weight loss, and abdominal mass [17, 19, 24, 25, 27, 28, 30, 31] which is similar to other research findings [36, 51–53].

Small lesions do complicate the diagnosis of renal carcinoid tumours as neither magnetic resonance imaging or computed tomography reliably distinguishes them from renal cell carcinoma [7]. Misdiagnosis of renal carcinoid tumour as RCC or Wilms tumour has been reported in approximately 15% of cases in the literature [38]. The clinical course of renal carcinoid is difficult to predict because of the rarity of the condition; however, it is largely believed to have an indolent course [54].

NETs of the kidney are capable of producing several hormones; however, the associations with neuroendocrine syndromes are notably rare [36]. NE syndromes like carcinoid syndrome, Cushing syndrome, Verner Morrison have been reported to occur in approximately 12.7% of patients with renal carcinoids [7, 55, 56].

### 4.5. Imaging

Ultrasound findings in renal carcinoid tumours as reported by Kim et al. which are consistent with previous reports demonstrate a hyper echoic mass with an incomplete hypo echoic or anechoic thin rim or halo and central or peripheral calcifications [44, 45, 51, 57, 58]. The most common imaging feature of all carcinoids tumours is calcification which is observed in 26.5% of primary renal carcinoids [7]. Central hypo echoic areas may suggest the presence of central necrosis [57].

The ultrasound findings alone pose a diagnostic dilemma because the findings in primary renal carcinoid tumour with or without teratoma are similar to findings in small renal cell carcinoma. They however differ from the findings in large renal cell carcinoma [45].

**Figure 1:**
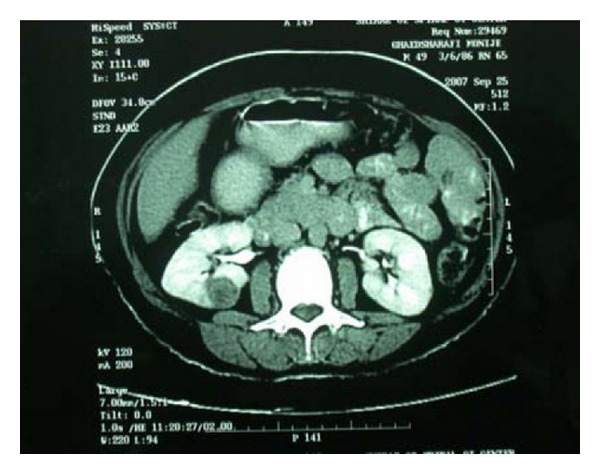
Demonstrating a well circumscribed tumour in the kidney. Reprinted from [19] with permission of the Editor-in-Chief of Saudi Journal of Kidney Diseases and Transplant on behalf of the editorial board.

Computed tomography findings may be heterogeneous demonstrating minimal contrast enhancement. It may also demonstrate a well-circumscribed enhanced or nonenhanced mass (Figure 1). The mass may be solid or with a cystic component with calcification in some patients [36, 44, 52, 53, 57]. Renal carcinoids have a characteristic tendency to appear with minimal or poor enhancement of contrast enhanced CT corresponding to the hypovascular or avascular lesions on angiography [36, 44, 52, 57].

Somatostatin receptor scintigraphy (SRS) is useful for diagnosis, staging, and monitoring after treatment for the development of recurrence or metastasis of carcinoid tumours. Radiolabeled octreotide is a somatostatin analogue that has a high affinity for somatostatin receptors. Primary carcinoids and metastatic lesions do have high affinity receptors for somatostatin in 87% of cases [59–61].

A study suggested that SRS is more sensitive, more specific, and more accurate than chromogranin A (CgA) levels (a recognized marker for carcinoid tumours) for metastatic evaluation of carcinoid tumours. Correlation of SRS with other imaging studies and clinical followup findings revealed a sensitivity, a specificity, and an accuracy of 82.9%, 97.7%, and 88.0%, respectively. Positive SRS correlated with elevation of serum CgA levels and both SRS and CgA should be considered to be useful tools in the evaluation of metastasis [62].

This is consistent with the findings of Mufarrij et al. who reported a case of a 40-year-old woman with primary renal carcinoid tumour with metastasis to the liver which was missed by CT scan and MRI scan was inconclusive. Four foci of increased radiotracer activity were identified by SRS [63]. Another case that highlights the importance of SRS was the reported case of a 67-year-old lady who had a right nephrectomy and retroperitoneal lymphadenectomy for suspected RCC which however turned out to be a carcinoid on further histology review. All imaging modalities failed to detect evidence of disease. SRS however detected SR positive tissue in the renal bed which was representative of residual carcinoid tumour [59].

It is important to note that the drawback for SRS is that normal renal uptake of the tracer material used in the evaluation of a primary renal carcinoid may obscure some lesions [7].

A comparative study of CT scans and SRS as imaging modalities for NETs concluded that both techniques are complementary, more importantly in patients with metastatic disease, inconclusive CT lesions or strong clinical or biochemical evidence of NET with negative scans [64].

### 4.6. Macroscopic Features

The size of primary carcinoid tumours of the kidney in our review ranges from 2 to 16 cm. Previous findings suggest a range from 1.5 to 30 cm [7]. These neoplasms with indolent course are usually diagnosed at a large size with approximately 75% greater than 4 cm. This could be due to the vacuous nature of the retroperitoneal space as kidneys are essentially retroperitoneal organs [7].

**Figure 2:**
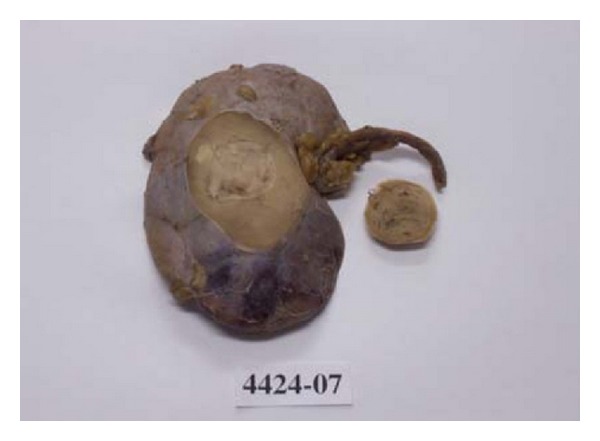
Macroscopic examination shows a well circumscribed renal tumour mass. Reprinted from [19] with permission of the Editor-in-Chief of Saudi Journal of Kidney Diseases and Transplant on behalf of the editorial board.

They are predominantly solid masses (Figure 2) which are unilateral and solitary with a third to be partially or wholly cystic. They have been reported to be well circumscribed with distinctly lobulated appearance. They exhibit a yellow orange to red tan colour of the cut surfaces [50, 65]. About half are confined to the kidney, a third involves perirenal fat, and approximately 10% involve the renal vein [53, 60, 66, 67].

Primary carcinoid tumour of the kidney thrombus involving the inferior vena cava has been reported [8]. Most cases arise in the renal parenchyma; however, rare cases arising from the renal pelvis have been reported [20, 36, 68, 69]. Calcification of varying sizes which may be central or peripheral [10–12, 16, 21–23, 25, 29, 51, 58], foci areas of haemorrhage [15, 30], necrosis and cystic changes has been documented. Although cases with necrosis have been previously reported [69–71] and also observed in our review [8, 15, 17, 26, 28], necrosis has been reported to be unusual [15, 36, 60, 67, 69]. The cystic changes may be uniloculated or multiloculated [34, 43, 53, 72]. Necrosis and foci areas of haemorrhage may be associated with neovascularization, rapid tumour growth, or feeding artery compression [73]. Calcification could possibly be related to long standing tumour growth as observed by Hamilton et al. in the case of a 64-year-old lady with a primary renal tumour that presented with watery-diarrhoea-hypokalaemia-achlorhydria (WDHA) syndrome [55]. Calcification may also be related to the presence of coexisting teratoma-like elements [10, 12, 44–46, 48]. Calcification seems to suggest a better prognosis while necrosis is associated with a higher incidence of metastasis; however, these relationships are not statistically significant [7].

### 4.7. Microscopic Features

Carcinoid tumours of the kidney share similar histological features as carcinoid tumour involving other sites in the body. They demonstrate distinct demarcations from the surrounding renal parenchyma. The most predominant histological pattern is the trabecular pattern; however, glandular, solid, nested, insular and mixed patterns have been reported [7, 36, 50].

The trabecular or ribbon like pattern mixed with nested pattern with peripheral palisading within the setting of a highly vascularized yet thin fibro-connective tissue stroma may be observed. Densely fibrotic or sclerotic stroma may also be seen [36, 38]. Foci of rosette like structures are characteristic of carcinoids. Mitotic activity though limited may be up to 4 mitotic figures per 10 high power fields (HPF).

**Figure 3:**
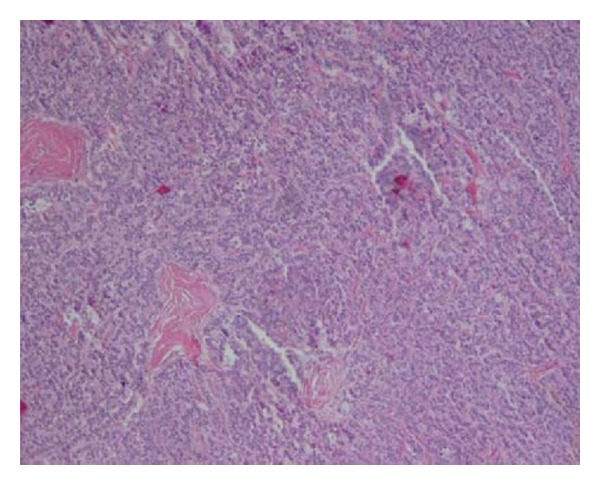
Microscopic section of the tumour showing tightly packed trabeculae (H & E ×250). Reprinted from [19] with permission of the Editor-in-Chief of Saudi Journal of Kidney Diseases and Transplant on behalf of the editorial board.

The neoplastic cells aremonomorphic round or polygonal with granular amphophilic to eosinophilic cytoplasm with indistinguishable cytoplasmic boundaries. The nuclei are round to oval, uniform in size, minimally pleomorphic, and with evenly distributed granular salt and pepper chromatin (Figure 3). Focal calcification with hyalinization and focal intraluminal mucin production may be seen [34, 36, 53, 67] and rarely metaplastic ossification has been reported [67, 69].

Distinct ultra-structural features of renal carcinoids include neoplastic cells with membrane bound electron dense neurosecretory granules ranging in size from 100 to 400 nm usually with a polar distribution. The cells have a swollen mitochondria with poorly developed rough endoplasmic reticulum and prominent Golgi complexes [34, 40, 41, 53, 55, 69, 72, 74].

The cell membranes are connected together by small but well developed desmosomes. Perinuclear aggregates of intermediate filaments have also been reported [7, 37, 38]. The aggregates of intermediate filaments are thought to represent abnormal accentuation of the small bundles of filaments normally present in kulchitsky type cells [75].

Huettner et al. opined that although these histological features are typical of carcinoid tumour and help in diagnosis they however give the tumour a pseudo papillary appearance [74]. 

### 4.8. Immunohistochemical Staining

**Figure 4:**
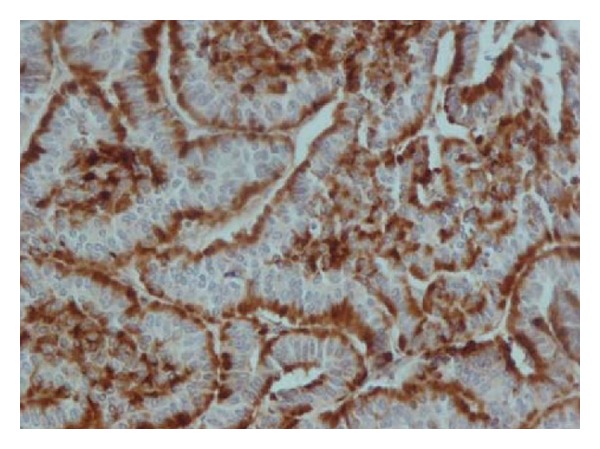
Diffuse staining of the cytoplasm with chromogranin. Reprinted from [19] with permission of the Editor-in-Chief of Saudi Journal of Kidney Diseases and Transplant on behalf of the editorial board.

These tumours have been reported to be immunoreactive to a variety of neuroendocrine markers. The tumours are commonly non-argentaffin and stains positively for Grimelius's agyrophil. They exhibit diffuse immunohistochemical staining for neuron-specific enolase, prohormone convertase (PC) 1/3 and PC 2, snare protein SNAP 25, vesicle associated membrane protein (VAMP), syntaxin, chromogranin (Figure 4), Leu-7, synaptophysin, and cytokeratins (broad-spectrum and low-molecular-weight) [34, 36–38, 52, 53, 60, 71, 76].

Serotonin, pancreatic polypeptide, vasoactive intestinal peptide, and somatostatin staining have been reported in some cases [34, 35, 37, 52, 53, 69]. Vimentin and S100 protein staining have been reported to be variable [7, 77]. It is recommended to perform immunochemistry with several antibodies when primary renal carcinoid is suspected [78]. Renal carcinoid tumours have demonstrated positivity for prostatic acid phosphatase; however, prostate specific antigen PSA is negative. This lends credence to the theory of a common hind gut origin for these tumours [37, 53, 60, 77, 79].

### 4.9. Grading

Differentiation suggests the extent to which neoplastic cells resemble their normal nonneoplastic counterparts while grade refers to the characteristic biological aggressiveness of the tumour. The WHO and ENETS (European Neuroendocrine Tumour Society) grading systems are based on the proliferative rates of the tumours.

The proliferative activity can be assessed by counting mitoses per unit area of the tumour usually expressed as mitoses per 10 high power field or as the percentage of immunostaining for the cell cycle-dependent proliferation marker Ki67 (MIB1) antigen [80–82].

To determine the Ki67 (MIB1) labeling index, 100 tumour cells have to be assessed in a hot-spot area. In case the Ki67 positivity is unevenly distributed, several tumour areas should be evaluated. The ENETS and WHO grading systems using the Ki67 index and mitotic count are G1 (<2% or <2 mitoses/10 HPF), G2 (3–20% or 2–20 mitoses/10 HPF), and G3 (>20% or >20 mitoses/10 HPF) [3, 80–82].

### 4.10. Metastasis

Although primary carcinoid tumour of the kidney shows a less aggressive course compared to RCC, metastasis is reportedly frequent. Metastatic disease is seen in 50%–60% of cases and is usually detected at initial evaluation [65]. Our review demonstrates that 44.8% of cases have metastatic disease.

The rate of metastasis is related to tumour size in renal carcinoids. Raslan et al. in their review reported that liver metastasis was evident either at presentation or during the course of the disease. The histologic features of the metastasis are similar to those of primary renal tumours [36]. Regional lymph nodes and the liver are the most frequent sites of metastasis [34] which is consistent with our review findings. Metastasis to other sites like the lungs breast and the thyroid has been reported [14, 17, 26, 27]. Distant spread has been reported for up to 7 years after nephrectomy which makes long-term followup care imperative [7, 36, 44, 70].

### 4.11. Treatment

Surgical resection of the kidney which may be open radical or partial nephrectomy with lymph node dissection has been variously reported as the treatment of choice in the management of localized primary renal carcinoid. The average followup time in our review is 20 months with 73.1% of patients without evidence of disease after surgical treatment which suggests that surgical treatment is curative.

Liver metastasis can be treated with open resection or with minimally invasive ablative procedures. Metastatic renal carcinoid has been noted to be resistant to chemotherapy [36, 83]. Studies with single agent therapy with 5FU, cisplatin and doxorubicin have been shown to have a response rate of approximately 20%, and combination chemotherapy does not seem to be significantly superior to single agents in the treatment of metastatic carcinoids [84].

### 4.12. Therapy with Somatostatin Analogues

Primary carcinoids and metastatic lesions do have high affinity receptors for somatostatin in 87% of cases [59–61]. It is also a known cytostatic agent to neoplastic cells [82]. Tumour response has strong correlations to somatostatin receptor expression.

The results of a placebo-controlled randomized double-blind prospective phase IIIB study on the effect of octreotide LAR on tumour growth in patients with metastatic midgut NETS (PROMID Study) suggest that octreotide LAR significantly lengthens time to tumour progression (14.3 months in octreotide group versus 6 months in the placebo group) in patients with functionally active and inactive NETs [85]. There are limited data in the literature regarding the use of somatostatin analogues in the treatment of primary NETs of the kidney. Progression-free survival period of 7 months with octreotide therapy combined with palliative radiotherapy was achieved in a patient with primary NET of the left kidney with metastatic disease. Patient however died within 11 months of diagnosis [17].

### 4.13. Targeted Therapy with Sunitinib or Everolimus

NETs of the gastrointestinal tract just like renal cell carcinoma have been reported to over express VEGF suggesting a possible target for antiangiogenic therapy [86]. Sunitinib is a multitargeted tyrosine kinase inhibitor that inhibits platelet derived growth factor (PDGF) receptor, VEGF receptor, and so forth. Everolimus, a more favourable pharmacologic derivative of rapamycin, inhibits the mammalian target of rapamycin (mTOR) pathway. mTOR inhibitors which are termed downstream multisignal inhibitors blocks receptor tyrosine kinases which are responsible for cell growth and proliferation, cell motility, and angiogenesis [87].

A phase II trial of everolimus combined with octreotide LAR in 60 patients with low grade to intermediate-grade mix of pancreatic (30) lung and gastrointestinal (30) NETs, demonstrated that everolimus was effective in the treatment of low grade and intermediate grade NET. 22% (13), 70% (42), and 22% (5) of patients achieved a partial remission (PR), stable disease (SD), and disease progression (DP), respectively [88]. There are limited data on the use of these novel approaches in the treatment of carcinoid tumours of the kidney. Sunitinib malate has been reportedly used as an adjuvant therapy with the patient disease free for 31 months. It may be argued that the patient was grossly disease-free following the surgery, and the role of sunitinib as an adjuvant in this patient remains unknown [16].

### 4.14. Peptide-Receptor Radionuclide Therapy

Lutetium DOTATATE alone or in combination with other agents has been used as a targeted therapy for NETs. Kwekkeboom et al. in a study of 35 patients with gastro-entero-pancreatic tumour reported complete remission in one patient (3%), partial remission in 12 (35%), stable disease in 14 (41%), and progressive disease in seven, (21%) 3 months after the final administration of therapy. Tumour response was positively correlated with a high uptake on the octreoscan and limited hepatic tumour mass [89]. Results of a controlled trial of 50 patients with disseminated NETs comparing the outcome of treatment with combination radioisotopes (^90^Y/^177^Lu-DOTATATE) and single radioisotope (^90^Y-DOTATATE) suggest that overall survival was significantly higher in the combination therapy group (*P* < 0.027) [90].

A phase II trial of ^177^Lutetium-DOTA-Octreotate therapy in somatostatin receptor-expressing NET is ongoing. 4 (30%) had a partial response to treatment and 9 patients (70%) exhibited stable disease among the 13 patients who have received two or more cycles of therapy. No disease progression was noted [91].

### 4.15. Prognosis

The prognosis of renal carcinoids is not predictable because they exhibit heterogeneous behaviour. Tumour stage at presentation appears to be the most important prognostic factor [36]. Our review showed 73.1% of patients without evidence of disease after treatment. This suggests an excellent prognosis.

Tumour size less than 4 cm and those confined to the renal parenchyma show a lower rate of metastasis and better prognosis. Age older than 40 was also found to have a significant relationship with advanced stage of disease at diagnosis and prognosis [7, 36]. Primary carcinoid tumour of the kidney that developed within the setting of horseshoe kidneys tends to have a more benign course even in the presence of lymph node involvement [34, 41, 70].

The prognosis of primary carcinoid tumour of the kidney arising within a mature teratoma appears good [10]. This however is in contrast to the findings of Romero et al. that show that neither horseshoe kidneys nor teratoma derived carcinoids had a better prognosis compared to primary carcinoid tumour originating from a normal kidney [7]. The number of mitoses and degree of cellular atypia appear to be important histological predictors of prognosis. There is no clear correlation between the histologic features of the disease and prognosis and tumour necrosis is not a predictor of prognosis [36, 44].

Gunes et al. reported the case of a 68-year-old male patient with a right renal mass lost to follow up without any medical intervention. Ten years later, the mass had nearly doubled in size with intrarenal spread, invasion of the precaval and paraaortic lymph nodes without distant metastasis. Right radical nephrectomy was done and a diagnosis of primary renal carcinoid tumour was made. This shows that localized renal carcinoid may remain stable and follow an indolent course without distant metastasis for as long as 10 years [92].

In conclusion, primary carcinoid tumours of the kidney are often well differentiated rare tumours with an indolent course treated with nephrectomy with excellent prognosis. They often pose diagnostic dilemmas because of their rarity, minimal awareness about them, and also because of similar presentation with other renal tumours which often leads to infrequent considerations by clinicians.

There is a paucity of data related to the treatment of primary kidney NETs with metastasis with novel approaches. Metastatic work up must always be done to rule out the possibility of metastasis from an occult tumour elsewhere when a clinical diagnosis of renal carcinoid is made. The use of complimentary imaging techniques including MRI, CT scan, and SRS must be encouraged. Diagnosis must be confirmed by the use of immunohistochemical stains. Long-term followup care is essential because of the prolonged course of disease despite metastasis.

## Figures and Tables

**Table 1:** Features of reviewed cases of primary carcinoid tumours of the kidney.

Cases	Age	Sex	Presentation	Size	Laterality	A/Pathology	Necrosis	Mitosis	Calcification	Haemorrhage	Metastasis	Treatment	F/U (mth)	Outcome
Szymanski et al. [8]	58	F	Total painless haematuria	7	R	−	+	−	−	−	−	RN	18	NED
Rafique [9]	44	F	Flank pain	8	L	−	−	scanty	−	−	−	RN	60	NED
Armah et al. [10]	50	F	Low back pain	9.7	R	HSK/MCT/PAC	−	−	+	−	−	PN	6	NED
Singh et al. [11]	57	M	Painless haematuria	8.5	L	−	−	−	+	−	LN	RN	12	NED
Armah and Parwani [12]	35	F	Flank pain	2	R	MT	−	−	+	−	−	PN	6	NED
Litwinowicz et al. [13]	66	M	Incidental	8	L	HSK	−	2	−	−	LN	RN	nr	nr
Hasteh et al. [14]	61	F	Nr	nr	L	nr	0	nr	Nr	Nr	liver, bone, breast	RN	60	AWD
Jain et al. [15]	40	F	loin pain	3.5	L	−	+	1	−	+	−	RN	60	NED
Finley et al. [16]	35	F	Abd pain	3.2	L	−	−	−	+	−	LN	RN	31	NED
Korkmaz et al. [17]	75	M	Flank pain, wt loss	8.5	L	−	+	4	−	−	LN, liver, lung, bone	Radiotherapy	11	DOD
Lane et al. [18]	68	F	Incidental	4.5	R	HSK	−	−	Nr	Nr	Liver	PN	28	DOD
Lane et al. [18]	36	F	Incidental	3.5	L	−	−	−	Nr	Nr	−	PN	12	NED
Lane et al. [18]	51	M	Incidental	8	R	HSK	−	−	Nr	Nr	−	RN	11	NED
Lane et al. [18]	43	F	Incidental	6	B	−	−	−	Nr	Nr	LN	Left RN right PN	6	NED
Lane et al. [18]	51	M	Flank pain, haematuria	3.5	L	HSK	−	−	Nr	Nr	−	RN	74	NED
Geramizadeh et al. [19]	49	F	Fever and flank pain	2.5	R	−	−	−	−	−	−	RN	3	NED
Kuroda et al. [20]	55	F	Incidental	5	L	−	−	0-1	−	−	−	RN	2	NED
Gedaly et al. [21]	45	F	Abd pain	8	R	−	−	nr	+	−	liver, LN	RN/hepatectomy	12	NED
Chiang et al. [22]	46	M	Flank dull ache	6.5	L	−	−	nr	+	−	LN, liver, bone	RN	12	AWD.
Sanjo et al. [23]	38	F	Incidental	6	R	−	−	low	+	−	−	RN	12	NED
La Rosa et al. [24]	39	M	Abd mass & haematuria	5.5	R	−	−	nr	−	−	LN	RN	3	AWD
Roy et al. [25]	34	M	Wt loss, epigastric discomfort	9.5	L	−	−	1	+	−	LN	RN	nr	nr
Bhalla et al. [26]	32	M	Abd pain	14.5	R	HSK	+	−	−	−	LN, thyroid	Nr	12	AWD
Rodríguez-Covarrubias et al. [27]	33	F	Low back pain, constipation	nr	B	HSK	−	low	−	−	liver, lung	Right RN/left PN	38	AWD
Ephrem et al. [28]	61	F	Abd pain wt loss	16	L	−	+	nr	−	−	−	RN/en bloc resection	1	NED
de Hoog et al. [29]	51	M	Lumbar back pain, sciatica	3	R	HSK	−	−	+	−	−	PN	12	NED
Chung et al. [30]	29	F	Urinary frequency & loin Pain	8	R	−	nr	low	−	+	−	RN	12	NED
Kubota et al. [31]	63	F	ABDOMINAL mass and fever	12	R	−	na	−	Na	+	−	RN	8	NED
Kawahara et al. [32]	50	M	Incidental	2.2	R	−	−	scanty	−	−	−	RN	ns	ns

AWD: alive with disease. NED: no evidence of disease. DOD: died of disease. NS: not stated. NR: not reported. RN/PN: radical/partial nephrectomy. HSK: horseshoe kidney. MT: mature teratoma. PAC: primary adenocarcinoma. MCT: mature cystic teratoma. LN: lymph node. mitotic figures counted per 10 HPF-high power field.

## References

[1] Lubarsch O (1888). Veber den primaren Krebs des Ileum nebst Bemerkingen über das gleichzeitige Vollkommen von Krebs und Tuberkulose. *Virchows Archiv*.

[2] Oberndorfer S (1907). Karzenoide tumoren des dunndarmes. *Frankfurter Zeitschrift für Pathologie*.

[3] Klimstra DS, Modlin IR, Coppola D, Lloyd RV, Suster S (2010). The pathologic classification of neuroendocrine tumors: a review of nomenclature, grading, and staging systems. *Pancreas*.

[4] Arnold R (2005). Introduction: definition, historical aspects, classification, staging, prognosis and therapeutic options. *Best Practice and Research*.

[5] Bosman FT, Carneiro F, Hruban R, Theise N (2010). *WHO Classification of Tumours of the Digestive System*.

[6] Modlin IM, Sandor A (1997). An analysis of 8305 cases of carcinoid tumours. *Cancer*.

[7] Romero FR, Rais-Bahrami S, Permpongkosol S, Fine SW, Kohanim S, Jarrett TW (2006). Primary carcinoid tumors of the kidney. *Journal of Urology*.

[8] Szymanski KM, Baazeem A, Sircar K, Tanguay S, Kassouf W (2009). Primary renal carcinoid tumour with inferior vena caval tumour thrombus. *Journal of the Canadian Urological Association*.

[9] Rafique M (2008). A primary carcinoid tumor of kidney. *Urology Journal*.

[10] Armah HB, Parwani AV, Perepletchikov AM (2009). Synchronous primary carcinoid tumor and primary adenocarcinoma arising within mature cystic teratoma of horseshoe kidney: a unique case report and review of the literature. *Diagnostic Pathology*.

[11] Singh PP, Malhotra AS, Kashyap V (2009). Carcinoid tumor of the kidney: an unusual renal tumor. *Indian Journal of Urology*.

[12] Armah HB, Parwani AV (2007). Primary carcinoid tumor arising within mature teratoma of the kidney: report of a rare entity and review of the literature. *Diagnostic Pathology*.

[13] Litwinowicz R, Szpor J, Januś G, Worek M, Okoń K (2011). Primary carcinoid tumour in horseshoe kidney. *Polish Journal of Pathology*.

[14] Hasteh F, Pu R, Michael CW (2007). A metastatic renal carcinoid tumor presenting as breast mass: a diagnostic dilemma. *Diagnostic Cytopathology*.

[15] Jain D, Sharma MC, Singh K, Gupta NP (2010). Primary carcinoid tumor of the kidney: case report and brief review of literature. *Indian Journal of Pathology and Microbiology*.

[16] Finley DS, Narula N, Valera VA (2011). Immunohistochemical basis for adjuvant anti-angiogenic targeted therapy for renal carcinoid: initial case report. *Urologic Oncology*.

[17] Korkmaz T, Seber S, Yavuzer D, Gumus M, Turhal NS (2013). Primary renal carcinoid: treatment and prognosis. *Critical Reviews in Oncology/Hematology*.

[18] Lane BR, Chery F, Jour G (2007). Renal neuroendocrine tumours: a clinicopathological study. *BJU International*.

[19] Geramizadeh B, Khezri A, Shariat M (2009). Renal carcinoid tumor.. *Saudi Journal of Kidney Diseases and Transplantation*.

[20] Kuroda N, Katto K, Tamura M (2008). Carcinoid tumor of the renal pelvis: consideration on the histogenesis. *Pathology International*.

[21] Gedaly R, Jeon H, Johnston TD, McHugh PP, Rowland RG, Ranjan D (2008). Surgical treatment of a rare primary renal carcinoid tumor with liver metastasis. *World Journal of Surgical Oncology*.

[22] Chiang M, Ou Y, Yang C, Cheng C, Ho H (2010). Primary renal carcinoid tumor with multiple metastases. *Journal of the Chinese Medical Association*.

[23] Sanjo H, Ito Y, Osaka K (2012). Renal carcinoid tumour: a case report. *Hinyokika Kiyo*.

[24] La Rosa FG, Flaig TW, Wilson S, Crawford ED, Kim FJ (2007). Sarcoidosis in a man with renal carcinoid tumor. *Oncology*.

[25] Roy S, Hooda S, Huang GJ, Pantanowitz L, Parwani AVA (2012). Novel case of concurrent renal tumours: chromophobe renal cell carcinoma and carcinoid tumor of the kidney with brief review of renal neuroendocrine tumours. *International Journal of Surgical Pathology*.

[26] Bhalla R, Popp A, Nassar A (2007). Case report: metastatic renal carcinoid to the thyroid diagnosed by fine needle aspiration biopsy. *Diagnostic Cytopathology*.

[27] Rodríguez-Covarrubias F, Gómez X, Valerio JC, Lome-Maldonado C, Gabilondo F (2007). Carcinoid tumor arising in a horseshoe kidney. *International Urology and Nephrology*.

[28] Ephrem O, Michael H, Jill C, Jack W (2009). Invasive neuroendocrine tumor of the kidney: a case report. *Rare Tumors*.

[29] de Hoog JP, Murray S, Chou W (2010). Horseshoe kidney and primary renal carcinoid tumour: a case report of a rare entity. *Grand Rounds*.

[30] Chung HY, Lau WH, Chu SM, Collins RJ, Tam PC (2007). Carcinoid tumour of the kidney in a Chinese woman presenting with loin pain. *Hong Kong Medical Journal*.

[31] Kubota Y, Seike K, Maeda S, Tashiro K (2010). A case of primary renal carcinoid tumor with hemorrhage. *Acta Urologica Japonica*.

[32] Kawahara T, Nagashima Y, Misaki H (2009). Primary renal carcinoid tumor with a mucinous cystadenoma element: letter to the editor. *International Journal of Urology*.

[33] Resnick ME, Unterberger H, McLoughlin PT (1966). Renal carcinoid producing the carcinoid syndrome. *Medical Times*.

[34] Krishnan B, Truong LD, Saleh G, Sirbasku DM, Slawin KM (1997). Horseshoe kidney is associated with an increased relative risk of primary renal carcinoid tumor. *Journal of Urology*.

[35] Van Den Berg E, Gouw ASH, Oosterhuis JW (1995). Carcinoid in a horseshoe kidney: morphology, immunohistochemistry, and cytogenetics. *Cancer Genetics and Cytogenetics*.

[36] Raslan WF, Ro JY, Ordonez NG (1993). Primary carcinoid of the kidney. Immunohistochemical and ultrastructural studies of five patients. *Cancer*.

[37] El-Naggar AK, Troncoso P, Ordonez NG (1995). Primary renal carcinoid tumor with molecular abnormality characteristic of conventional renal cell neoplasms. *Diagnostic Molecular Pathology*.

[38] Unger PD, Russell A, Thung SN, Gordon RE (1990). Primary renal carcinoid. *Archives of Pathology and Laboratory Medicine*.

[39] Guy L, Bégin LR, Oligny LL, Brock GB, Chevalier S, Aprikian AG (1999). Searching for an intrinsic neuroendocrine cell in the kidney. *Pathology Research and Practice*.

[40] Cauley JE, Almagro UA, Jacobs SC (1988). Primary renal carcinoid tumor. *Urology*.

[41] Bégin LR, Guy L, Jacobson SA, Aprikian AG (1998). Renal carcinoid and horseshoe kidney: a frequent association of two rare entities-a case report and review of the literature. *Journal of Surgical Oncology*.

[42] Motta L, Candiano G, Pepe P, Panella P, Galia A, Aragona F (2004). Neuroendocrine tumor in a horseshoe kidney: case report and updated follow-up of cases reported in the literature. *Urologia Internationalis*.

[43] Kojiro M, Ohishi H, Isobe H (1976). Carcinoid tumor occurring in cystic teratoma of the kidney. A case report. *Cancer*.

[44] Yoo J, Park S, Lee HJ, Kang SJ, Kim BK (2002). Primary carcinoid tumor arising in a mature teratoma of the kidney: a case report and review of the literature. *Archives of Pathology and Laboratory Medicine*.

[45] Kim J, Suh K (2004). Primary carcinoid tumor in a mature teratoma of the kidney: ultrasonographic and computed tomographic findings. *Journal of Ultrasound in Medicine*.

[46] Kurzer E, Leveillee RJ, Morillo G (2005). Rare case of carcinoid tumor arising within teratoma in kidney. *Urology*.

[47] Fetissof F, Benatre A, Dubois MP (1984). Carcinoid tumor occurring in a teratoid malformation of the kidney. An immunohistochemical study. *Cancer*.

[48] Lodding P, Hugosson J, Hansson G (1997). Primary carcinoid tumour with ossification masquerading as calyx stone in a horseshoe kidney. *Scandinavian Journal of Urology and Nephrology*.

[49] Shibata R, Okita H, Shimoda M (2003). Primary carcinoid tumor in a polycystic kidney. *Pathology International*.

[50] Murali R, Kneale K, Lalak N, Delprado W (2006). Carcinoid tumors of the urinary tract and prostate. *Archives of Pathology and Laboratory Medicine*.

[51] McKeown DK, Nguyen GK, Rudrick B, Johnson MA (1988). Carcinoid of the kidney: radiologic findings. *American Journal of Roentgenology*.

[52] Juma S, Nickel JC, Young I (1989). Carcinoids of the kidney: case report and literature review. *Canadian Journal of Surgery*.

[53] Schlussel RN, Kirschenbaum AM, Levine A, Unger P (1993). Primary renal carcinoid tumor. *Urology*.

[54] Cole EE, DeSouza R, Shappell S, Cookson MS (2004). Primary renal carcinoid tumor. *Journal of Urology*.

[55] Hamilton I, Reis L, Bilimoria S, Long RG (1980). A renal vipoma. *British Medical Journal*.

[56] Hannah J, Lippe B, Lai-Goldman M, Bhuta S (1988). Oncocytic carcinoid of the kidney associated with periodic Cushing’s syndrome. *Cancer*.

[57] Moulopoulos A, DuBrow R, David C, Dimopoulos MA (1991). Primary renal carcinoid: computed tomography, ultrasound, and angiographic findings. *Journal of Computer Assisted Tomography*.

[58] Stahl RE, Sidhu GS (1983). Primary carcinoid of the kidney: a light and electron microscopic and immunochemical study. *Journal of Urology*.

[59] McCaffrey JA, Reuter V, Herr HW, Macapinlac HA, Russo P, Motzer RJ (2000). Carcinoid tumor of the kidney: the use of somatostatin receptor scintigraphy in diagnosis and management. *Urologic Oncology*.

[60] Eble JN, Sauter G, Epstein JI, Sesterhenn IA (2004). *World Health Organization Classification of Tumors. Pathology and Genetics of Tumours of the Urinary System and Male Genital Organs*.

[61] Reubi JC, Kvols LK, Waser B (1990). Detection of somatostatin receptors in surgical and percutaneous needle biopsy samples of carcinoids and islet cell carcinomas. *Cancer Research*.

[62] Namwongprom S, Wong FC, Tateishi U, Kim EE, Boonyaprapa S (2008). Correlation of chromogranin A levels and somatostatin receptor scintigraphy findings in the evaluation of metastases in carcinoid tumors. *Annals of Nuclear Medicine*.

[63] Mufarrij P, Varkarakis IM, Studeman KD, Jarrett TW (2005). Primary renal carcinoid tumor with liver metastases detected with somatostatin receptor imaging. *Urology*.

[64] King CMP, Reznek RH, Bomanji J (1993). Imaging neuroendocrine tumours with radiolabelled somatostatin analogues and X-Ray computed tomography: A Comparative Study. *Clinical Radiology*.

[65] Fine SW (2007). Neuroedocrine lesions of the genitourinary tract. *Advances in Anatomic Pathology*.

[66] Canacci AM, MacLennan GT (2008). Carcinoid tumor of the kidney. *Journal of Urology*.

[67] McVey RJ, Banerjee SS, Eyden BP, Reeve RS, Harris M (2002). Carcinoid tumor originating in a horseshoe kidney. *In Vivo*.

[68] Ji X, Li W (1994). Primary carcinoid of the renal pelvis. *Journal of Environmental Pathology, Toxicology and Oncology*.

[69] Rudrick B, Nguyen GK, Lakey WH (1995). Carcinoid tumor of the renal pelvis: report of a case with positive urine cytology. *Diagnostic Cytopathology*.

[70] Quinchon JF, Aubert S, Biserte J, Lemaitre L, Gosselin B, Leroy X (2003). Primary atypical carcinoid of the kidney: a classification is needed. *Pathology*.

[71] Pohar-Marinšek Ž (1993). Primary carcinoid of the kidney. *International Urology and Nephrology*.

[72] Toker C (1974). Carcinoidal renal tumor. *Journal of Urology*.

[73] Takeshima Y, Inai K, Yoneda K (1996). Primary carcinoid tumor of the kidney with special reference to its histogenesis. *Pathology International*.

[74] Huettner PC, Bird DJ, Chang YC, Seiler MW (1991). Carcinoid tumor of the kidney with morphologic and immunohistochemical profile of a hindgut endocrine tumor: report of a case. *Ultrastructural Pathology*.

[75] McDowell EM, Barrett LA, Trump BF (1976). Observations on small granule cells in adult human bronchial epithelium and in carcinoid and oat cell tumors. *Laboratory Investigation*.

[76] Uribe-Uribe NO, Gu X, Herrera GA (2006). Primary renal carcinoid tumor. *Pathology Case Reviews*.

[77] Goldblum JR, Lloyd RV (1993). Primary renal carcinoid: case report and literature review. *Archives of Pathology and Laboratory Medicine*.

[78] Osamura RY, Kumaki N, Kajiwara H (2002). Immnochemistry and electron microscopy for the diagnosis of neuro endocrine tumours. *Pathology Case Reviews*.

[79] Bégin LR, Jamison BM (1993). Renal carcinoid: a tumor of probable hindgut neuroendocrine phenotype. *Journal of Urologic Pathology*.

[80] Rindi G, Klöppel G, Ahlman H (2006). TNM staging of foregut (neuro)endocrine tumours: a consensus proposal including a grading system. *Virchows Archiv*.

[81] Rindi G, Klöppel G, Couvelard A (2007). TNM staging of midgut and hindgut (neuro) endocrine tumors: a consensus proposal including a grading system. *Virchows Archiv*.

[82] Schally AV (1988). Oncological applications of somatostatin analogues. *Cancer Research*.

[83] Ribeiro JC, Sousa L, Santos R (2010). Primary neuroendocrine tumor of the kidney. *Actas Urologicas Espanolas*.

[84] Khan MU, Coleman RE (2008). Diagnosis and therapy of carcinoid tumors-current state of the art and future directions. *Nuclear Medicine and Biology*.

[85] Rinke A, Müller H, Schade-Brittinger C (2009). Placebo-controlled, double-blind, prospective, randomized study on the effect of octreotide LAR in the control of tumor growth in patients with metastatic neuroendocrine midgut tumors: a report from the PROMID study group. *Journal of Clinical Oncology*.

[86] Turner HE, Harris AL, Melmed S, Wass JAH (2003). Angiogenesis in Endocrine Tumors. *Endocrine Reviews*.

[87] Grozinsky-Glasberg S, Franchi G, Teng M (2008). Octreotide and the mTOR inhibitor RAD001 (everolimus) block proliferation and interact with the Akt-mTOR-p70S6K pathway in a neuro-endocrine tumour cell line. *Neuroendocrinology*.

[88] Yao JC, Phan AT, Chang DZ (2008). Efficacy of RAD001 (everolimus) and octreotide LAR in advanced low- to intermediate-grade neuroendocrine tumors: results of a phase II study. *Journal of Clinical Oncology*.

[89] Kwekkeboom DJ, Bakker WH, Kam BL (2003). Treatment of patients with gastro-entero-pancreatic (GEP) tumours with the novel radiolabelled somatostatin analogue [177Lu-DOTA0,Tyr3]octreotate. *European Journal of Nuclear Medicine and Molecular Imaging*.

[90] Kunikowska J, Królicki L, Hubalewska-Dydejczyk A, Mikolajczak R, Sowa-Staszczak A, Pawlak D (2011). Clinical results of radionuclide therapy of neuroendocrine tumours with90Y-DOTATATE and tandem90Y/177Lu-DOTATATE: which is a better therapy option?. *European Journal of Nuclear Medicine and Molecular Imaging*.

[91] 177Lutetium-DOTA-Octreotate therapy in Somatostatin Receptor-Expressing Neuroendocrine Neoplasms. http://www.clinicaltrials.gov/.

[92] Gunes A, Yilmaz U, Ugras M, Mizrak B (2002). Primary renal carcinoid natural history of the disease for ten years: casereport. *BMC Urology*.

